# Higher prevalence and gene amplification of HPV16 in oropharynx as compared to oral cavity

**DOI:** 10.1590/1678-775720160009

**Published:** 2016

**Authors:** Hideo SHIGEISHI, Masaru SUGIYAMA, Kouji OHTA, Mohammad Zeshaan RAHMAN, Masaaki TAKECHI

**Affiliations:** 1 - Hiroshima University, Institute of Biomedical and Health Sciences, Department of Oral and Maxillofacial Surgery, Hiroshima, Japan.; 2 - Hiroshima University, Institute of Biomedical and Health Sciences, Department of Public Oral Health, Hiroshima, Japan.

**Keywords:** HPV16, Oral cavity, Oropharynx, PCR, Gene amplification

## Abstract

**Objective:**

The objective of this study was to clarify differences regarding HPV16 infection and gene amplification between the oral cavity and oropharynx in healthy individuals.

**Material and Methods:**

The subjects were 94 healthy asymptomatic individuals (41 males, 53 females; mean age 58.6 years, range 16-97 years) who visited the Department of Oral and Maxillofacial Reconstructive Surgery of the Hiroshima University Hospital from 2014 to 2015. Oral epithelial cells were collected from oral rinse and pharynx gargle samples and placed in saline. The human endogenous retrovirus gene ERV3-1 was used as a reference to estimate the number of human cells in each sample. DNA samples were extracted from approximately 10,000 human cells and tested for HPV16 DNA by PCR using a type-specific primer. Similarly, we analyzed the HPV16 viral copy number in HPV16-positive cases using real-time PCR to examine genomic amplification.

**Results:**

The percentage of HPV16-positive cases was higher in the gargle (28.7%) as compared to the rinse (16.0%) samples. In the oral rinse samples, males (26.8%) showed a significantly higher rate of HPV16 than females (7.5%) (P=0.021). Importantly, in older subjects (aged 60-89 years), gargle samples showed a significantly higher rate of HPV16 (33.3%) than oral rinse samples (13.7%) (P=0.034). The average number of viral copies was approximately 8 times higher in the gargle than in the oral rinse samples (0.16±0.27 vs. 1.35±1.26 copy numbers per cell), a significant difference (P<0.001).

**Conclusion:**

Our findings suggest that the oropharynx is more susceptible to HPV16 infection as compared to the oral cavity, while HPV16 gene amplification is also more commonly found in the oropharynx.

## INTRODUCTION

The human papilloma virus (HPV) consists of a circle of double-stranded DNA and belongs to the family of papillomaviridae, with infection generally occurring in the skin and mucosa^7,8^. HPV infection remains in basal cells, though is discharged outside the body upon differentiation of the infected cells into keratinocytes^8^. The virus, therefore, does not enter the bloodstream and is able to evade immune surveillance. The HPV genome consists of six early open reading frames (ORFs) (E1, E2, E4, E5, E6, E7), two late ORFs (L1, L2), and a non-coding long control region (LCR)^7,8^. According to the International Agency for Research on Cancer (IARC), 12 HPV types (HPV16, 18, 31, 33, 35, 39, 45, 51, 52, 56, 58, 59) are defined as Group 1 human carcinogens^3^, of which HPV16 was identified as the cause of cervical cancer in the early 1980s and considered to be the most important risk factor for development of cervical cancer^30^. In addition, persistent infection with oral HPV16 is also thought to drive the development of oropharyngeal cancer^12^.

We previously reported that most precancerous lesions caused by HPV16 infection of the oral cavity finally progress to oral squamous cell cancer, indicating that oral HPV16 infection is significantly associated with malignant transformation of precursor lesions^24^. However, few epidemiologic studies have focused on oral HPV16 prevalence in healthy Japanese individuals and that has not been fully elucidated. In this study, we investigated oral rinse and gargle samples obtained from healthy individuals to clarify differences of HPV16 infection and gene amplification between the oral cavity and oropharynx.

## MATERIAL AND METHODS

### Subjects

The subjects of this study were 94 healthy asymptomatic individuals (41 males, 53 females; mean age 58.6 years, range 16-97 years), who visited the Department of Oral and Maxillofacial Reconstructive Surgery of the Hiroshima University Hospital from 2014 to 2015. None had evidence of oral cancer or pre-malignant lesions. The study design was approved by the Ethics Committee of the Hiroshima University and all participants signed an informed consent form.

### Oral rinse and gargle sample processing and DNA extraction

Oral rinse samples were used to evaluate HPV16 infection in the oral cavity while gargle samples were used to the evaluation of the oropharynx. Exfoliated epithelial cells were collected in samples obtained by a 30-second oral rinse and a separate 30-second gargle^9^. Briefly, subjects were asked to first rinse their mouth with 10 mL of saline for 30 seconds and then spit into a sterile 50-mL falcon tube. Next, they gargled 10 mL of saline for another 30 seconds and spit that sample into a different 50-mL falcon tube. Immediately after collection, all samples were centrifuged at 3000x *g* for 10 minutes at 4°C, then the supernatant was decanted, the pellet re-suspended in 10 ml of phosphate-buffered saline (PBS), and centrifugation repeated. Thereafter, the pellets were stored at -80°C until further processing. Finally, DNA was extracted and purified using a Wizard^**®**^ Genomic DNA Purification Kit.

### Quantitation of human cell numbers

**Figure 1:**
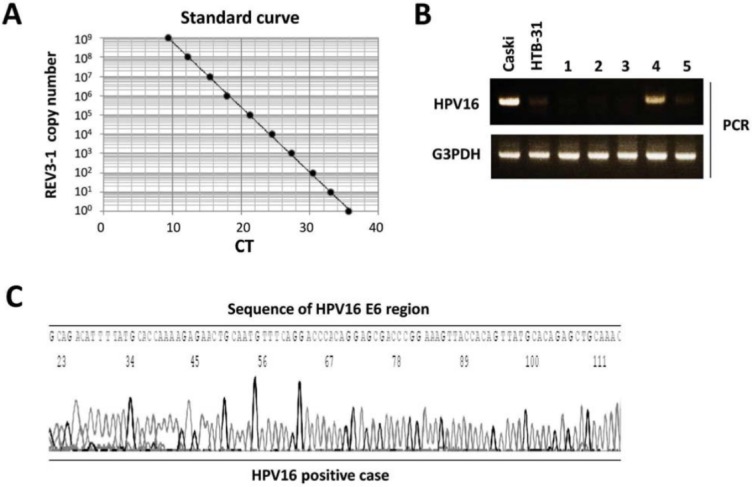
Detection of HPV16 by PCR. (A) Standard curve indicating CT value vs. ERV3-1 copy number. A standard curve was calculated by plotting obtained CT values against serial 10-fold dilutions produced with ERV3-1 copy numbers ranging from 100 to 109. (B) Agarose gel electrophoresis of amplified PCR products from cell lines and oral samples. Ten microliters of each 20-μl PCR product were separated on a 3.0% agarose gel. HPV16 DNA was detected in Caski cells (HPV16 positive), but not in HTB-31 cells (HPV negative). Lanes: 1-5, oral rinse samples (C) PCR products were examined by sequencing for the HPV16 E6 region

DNA amounts extracted from the oral rinse samples were not equivalent to the amount of human DNA, due to the quantity of non-human derived DNA. Therefore, we employed the human endogenous retrovirus group 3 member 1 (ERV3-1) gene to quantitate human cells using a real-time PCR assay, according to a previously reported method^9^. A 1.7-kb Hind III:Pst I fragment within the ERV3-1 genome was prepared and then cloned into a 2.7-kb pUC57 vector. Serial 10-fold dilutions of the pUC57 vector were made with copy numbers ranging from 10^0^ to 10^9^. Calculation of number of copies was performed based on the DNA molecular weight of the plasmid, according to a previously reported method^29^. Next, we performed real-time PCR assays on the diluted samples using an ERV3-1-specific primer. Quantitation of DNA levels was done using a CFX connect real-time PCR detection system (BioRad, Hercules, CA, USA) and SYBR Green PCR Master Mix (TOYOBO, Tokyo, Japan), with a reaction mixture containing 1.0 µl of DNA, 12.5 µl of SYBR Green Mix, and 10 µmol of each pair of oligonucleotide primers. The primer sequences were as follows: ERV3-1; 5’- CATGGGAAGCAAGGGAACTAATG-3’ (sense) and 5’- CCCAGCGAGCAATACAGAATTT -3’ (antisense)^9^. Amplifications were performed with an initial melting at 95°C for 5 minutes, then 40 cycles of denaturing at 95°C for 30 seconds, annealing at 55°C for 30 seconds, and extension at 72°C for 1 minute. A standard curve indicating CT value versus ERV3-1 copy number was obtained (Figure 1A). The number of cells, determined by use of the ERV3-1 diploid genome, was used to estimate the number of human cells in each sample.

### HPV16 DNA detection by PCR

The HPV16 sequence was examined by PCR with type-specific primers derived from the E6 open reading frame using a PCR HPV Detection Set (Takara, Osaka, Japan). The primers amplified a fragment with a length of 140 base pairs. Samples were tested for G3PDH to confirm collection of an oral cellular sample and those without G3PDH were excluded from analysis. A positive control containing HPV16 DNA extracted from Caski cells and a negative control containing DNA purified from the HPV-negative cell line HTB31 were used for the PCR reactions. We prepared DNA samples extracted from approximately 10,000 human cells in each PCR mixture. Each of those mixtures was amplified with 10x PCR buffer (TOYOBO), dNTPs, Taq DNA polymerase, and HPV16 primers. The primer sequences for *HPV16* were 5’- AAGGGCGTAACCGAAATCGGT-3’ (sense) and 5’-GTTTGCAGCTCTGTGCATA-3’ (antisense), and those for *G3PDH* were 5’- ACCACAGTCCATGCCATCAC-3’ (sense) and 5’-TCCACCACCCTGTTGCTGTA-3’ (antisense). The PCR program included initial melting at 95°C for 5 minutes, followed by 28 cycles at 95°C for 30 seconds, 55°C for 2 minutes, and 72˚C for 30 seconds. After the reaction, 10 μL of the PCR product was electrophoresed on 3% agarose gels with ethidium bromide staining. To confirm the HPV16 DNA sequence, we performed sequencing for HPV16. After PCR was completed, 10 μl of the PCR product was analyzed by electrophoresis using 2% agarose gels. For the sequencing reaction, a BigDye Terminator v3.1 cycle sequencing kit (Applied Biosystems, Foster City, CA, USA) was used. Amplified DNA fragments were sequenced with an ABI PRISM 310 genetic analyzer (Applied Biosystems).

### HPV16 viral copy quantitation

Real-time PCR assays were performed to determine the viral load of HPV16 DNA in the samples. We prepared a 200-bp fragment within the HPV16 E6 genome, which was then cloned into a 2.7-kb pUC57 vector. Serial 10-fold dilutions of the vector were made with copy numbers ranging from 10^0^to 10^9.^ Serial 10-fold dilutions were used to generate a standard curve, which indicated the CT value versus the copy number of HPV16 E6. We then prepared DNA samples extracted from approximately 10,000 human cells from each PCR mixture. Real-time PCR was performed using a type-specific primer with a PCR HPV Detection Set (Takara). The PCR program was as follows: initial melting at 95°C for 5 minutes, followed by 40 cycles at 95°C for 30 seconds, 57°C for 2 minutes, and 72°C for 30 seconds. A standard curve was used to enumerate the copy number of HPV16 in each sample.

### Statistical analysis

Fisher’s exact test was used to examine the correlation between oral HPV16 infection and clinical parameters. Student’s t-test was used to evaluate significant differences regarding HPV16 copy numbers. *P* values less than 0.05 were regarded as statistically significant.

## RESULTS

### HPV16 prevalence in oral rinse and pharynx gargle samples

HPV16 prevalence was examined in a total of 94 oral rinse and gargle samples. The product of HPV16 DNA was detected in Caski cells (HPV16 positive), but not in HTB-31 cells (HPV negative), by PCR analysis (Figure 1B). Next, the PCR products were confirmed by DNA sequencing to be those of HPV16 (Figure 1C). We found HPV16-positive samples in 15 (16.0%) oral rinse samples and 27 (28.7%) gargle samples, showing a higher prevalence in the gargle samples. There were only 8 subjects (8.5%) with both HPV16-positive oral rinse and gargle samples, whereas 19 (20.2%) were HPV16 positive in the gargle sample alone.

### Correlation between HPV16 infection and clinical factors

Males showed a higher rate of HPV16 prevalence than females (26.8% vs. 7.5%) in the oral rinse samples (Table 1). Of the 11 oral HPV16-positive men, 4 were denture users and 4 were young (18-29 years old), with a significant correlation between HPV16 infection and male gender found (P=0.021). As for age, subjects aged 20-29 years old showed a high level of HPV16 prevalence (26.8%) in the oral rinse samples. On the other hand, the 30-39, 60-69, and 80-89 year-old groups exhibited high HPV16 prevalence (37.5%, 44.4%, and 37.5%, respectively) in the gargle samples. We also performed statistical analysis to compare young (20-59 years old) with older (60-89 years old) subjects (Table 2). HPV16 prevalence was higher in the gargle samples as compared to the oral rinse samples in both of those groups. However, the odds ratio for a HPV16-positive gargle sample was higher in the older group (odds ratio, 3.14 vs. 1.37). Furthermore, the gargle samples showed a significantly higher rate of HPV16 (33.3%) than the oral rinse samples (13.7%) in that older group (P=0.034). In addition, subjects who used dentures showed an increased percentage of HPV16 infection in both oral rinse and gargle samples as compared to non-denture users (oral rinse, 20.7% vs. 13.4%; gargle, 34.5% vs. 25.3%). However, we found no significant correlation between HPV16 infection and denture use in either the oral rinse or gargle samples.

**Table 1:** Correlation between oral HPV16 infection and clinical parameters

	Rinse sample			Gargle sample		
Clinical characteristics	HPV16 (-)	HPV16 (+)	P value	HPV16 (-)	HPV16 (+)	P value
**Gender**						
Male (41)	30 (73.2%)	11 (26.8%)	0.021	27 (65.9%)	14 (34.1%)	0.36
Female (53)	49 (92.5%)	4 (7.5%)		40 (75.5%)	13 (24.5%)	
**Age in years**						
10-19 (3)	2 (66.7%)	1 (33.3%)	0.49	2 (66.7%)	1 (33.3%)	0.61
20-29 (14)	10 (71.4%)	4 (28.6%)		12 (85.7%)	2 (14.3%)	
30-39 (8)	6 (75.0%)	2 (25.0%)		5 (62.5%)	3 (37.5%)	
40-49 (7)	6 (85.7%)	1 (14.3%)		5 (71.4%)	2 (28.6%)	
50-59 (9)	9 (100%)	0 (0.0%)		7 (77.8%)	2 (22.2%)	
60-69 (18)	14 (77.8%)	4 (22.2%)		10 (55.6%)	8 (44.4%)	
70-79 (17)	15 (88.2%)	2 (11.8%)		14 (82.4%)	3 (17.6%)	
80-89 (16)	15 (93.8%)	1 (6.3%)		10 (62.5%)	6 (37.5%)	
≥90 (2)	2 (100.0%)	0 (0.0%)		2 (100.0%)	0 (0.0%)	
**Denture use**						
(-) (65)	56 (86.2%)	9 (13.8%)	0.54	48 (73.8%)	17 (26.2%)	0.46
(+) (29)	23 (79.3%)	6 (20.7%)		19 (65.5%)	10 (34.5%)	

Fisher’s exact test was used for statistical analysis. P values less than 0.05 were regarded to be statistically significant

**Table 2:** Correlation between oral HPV16 infection and sample type among young adults and older people

	Age 20-59 (n=38)				Age 60-89 (n=51)			
Sample type	HPV16 (-)	HPV16 (+)	OR (95%CI)	P value	HPV16 (-)	HPV16 (+)	OR (95%CI)	P value
**Rinse sample**	31 (81.6%)	7 (18.4%)	1.37 (0.47-4.05)	0.78	44 (86.3%)	7 (13.7%)	3.14 (1.19-8.24)	0.034
**Gargle sample**	29 (76.3%)	9 (23.7%)			34 (66.7%)	17 (33.3%)		

Fisher’s exact test was used for statistical analysis. P values less than 0.05 were regarded to be statistically significant. OR: Odds ratio; 95%CI: 95% Confidence interval

### Quantitation of HPV16 viral copy number using real-time PCR

**Figure 2:**
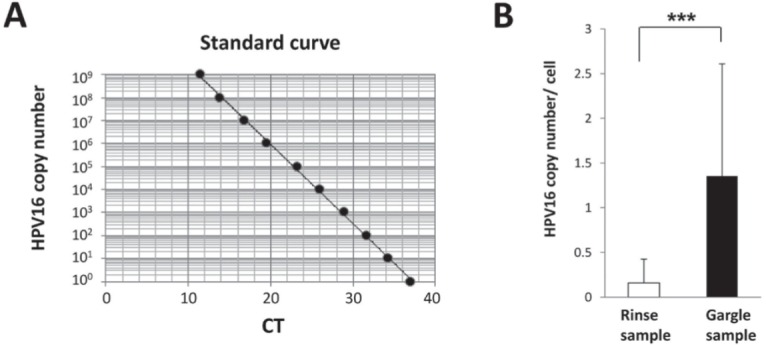
Quantification of HPV16 viral copy number by real-time PCR. (A) Standard curve indicating CT value vs. HPV16 copy number. A standard curve was calculated by plotting obtained CT values against serial 10-fold dilutions produced with HPV16 copy numbers ranging from 100 to 109. (B) The average number of viral copies was significantly higher in the gargle samples as compared to the oral rinse samples (0.16±0.27 vs. 1.35±1.26 *per* cell) (***P<0.001)

In HPV16-positive cases, we analyzed the HPV16 viral copy number using real-time PCR to examine genomic amplification. Serial 10-fold dilutions of a plasmid vector containing HPV16 E6 DNA were used to generate a standard curve (Figure 2A), then we evaluated the number of HPV16 DNA copies as copy number *per* human cell. The average number of viral copies was approximately 8 times higher in the gargle samples than in the oral rinse samples (0.16±0.27 vs. 1.35±1.26 copy numbers per cell) (Figure 2B). We found a significant difference regarding HPV16 E6 DNA copy number between the gargle and oral rinse samples (P<0.001). These results suggest that HPV16 gene amplification is more commonly found in the oropharynx as compared to the oral cavity.

## DISCUSSION

Oral HPV infection is considered to be transmitted through sexual behavior, thus marital status, number of sex partners, oral sex, and anal sex are thought to be related variables. Oral HPV prevalence is significantly higher in individuals who have participated in oral sex as compared to those who have not, indicating that as a risk factor for oral HPV infection^11^. As for other risk factors, smoking and poor oral hygiene related to such variables as periodontitis, gingivitis, and denture use have been found to be associated with oral HPV infection^12,15,18^.

As for smoking, the chemical substances contained in tobacco may be involved and suppress immune mechanisms in the oral cavity^11^, while the relationship of denture use to HPV infection suggests that oral mucosal injury caused by mechanical stimulation from denture wearing and poor oral hygiene due to unclean dentures may have an association^23^. Thus, HPV infection may be associated with oral environmental changes due to local effects, such as smoking and denture use. Importantly, our results showed that being male is a significant risk factor for oral HPV16 infection. Although the reasons remain unclear, smoking, denture use, sexual activity, and other factors may be related to the high rate of oral infection seen in men.

In the subjects of this study, the prevalence rate of HPV16 was higher in the gargle samples as compared to the oral rinse samples. Gargle samples from the older groups also showed significantly higher rates of HPV16 prevalence than oral rinse samples from those subjects, indicating an important association between age and HPV16 infection in the oropharyngeal region, and that age distribution is likely to be a cause of differences between oral rinse and gargle samples. In addition, HPV16 copy numbers were significantly elevated in the gargle samples. Despite cross-mixing of the samples between the oral cavity and oropharyngeal region, we considered that the collected oral rinse and gargle samples were associated with HPV DNA present primarily in the oral cavity and oropharyngeal region, respectively. Thus, our observations suggest that the oropharynx is more susceptible to HPV16 infection and HPV16 gene amplification is more commonly found there as compared to the oral cavity.

In a previous study of head and neck cancer cases, the positive rate of HPV was 10% in patients with oral cavity cancer, 83.2% in those with oropharyngeal cancer, and 44.4% in those with nasopharynx cancer, demonstrating the highest prevalence in oropharyngeal cancer cases, while approximately 90% of the HPV-positive oropharyngeal cancer patients also showed HPV16 infection^27^. Furthermore, Wang, et al.^28^ (2012) reported that male oropharyngeal cancer patients showed a higher rate of HPV16 infection than female patients. Our results also showed that the gargle specimens from males had a higher rate of HPV16 infection as compared to those from females. Another study also reported that the HPV16 genome copy number was much higher in oropharyngeal cancer than oral cancer specimens^22^. Together, these results support the notion that the oropharynx is a more common site for HPV16 infection as compared to other head and neck regions. In addition, the variability of HPV16 infection rate among different sites (i.e., oral, oropharyngeal, laryngeal) suggests the biological characteristics of HPV-related cancer.

Findings for the prevalence of oral HPV vary substantially among reported studies, which has been attributed to differences in study populations, as well as approaches for specimen collection and detection methods^1,2,4-6,13,14,16,17,19-21,25,26^. In recent studies of oral HPV infection, the methods most frequently used for oral sample collection have included oral swabbing, oral rinsing, and gargling with mouthwash or saline, with rinsing with mouthwash or saline thought to be highly effective. PCR assays targeting the HPV L1 region have also been commonly performed in many studies, while direct HPV genotyping without HPV detection was utilized in some^1,2,4-6,13,14,16,17,19-21,25,26^. In those, HPV genotypes were determined by PCR using type-specific primers, hybridization of amplified HPV DNA with type-specific probes, or sequencing, with DNA hybridization frequently using the LINEAR ARRAY HPV Genotyping Test , as it is useful for detection of combined HPV infection, though less sensitive and positive rates may vary with different cut-off values^10^. Thus, we used a combination of PCR with type-specific primers and sequencing for HPV16 detection to accurately determine HPV16 infection status in the present subjects.

Taken together, our observations indicate that HPV16 infection has a higher rate of prevalence in gargle samples. Thus, HPV16 is considered to be an important risk factor for development of head and neck cancer, especially oropharyngeal cancer. Future studies are needed to examine the risks of smoking, oral hygiene, and sexual behavior in relation to HPV infection, and will likely lead to a decrease in oral HPV infection rates, while elucidation of the molecular biology of HPV may reduce the occurrence of HPV-related head and neck cancer.

## CONCLUSION

Our results indicate that the oropharynx is more susceptible to HPV16 infection as compared to the oral cavity, while HPV16 gene amplification is also more commonly found in the oropharynx.
